# Gene Regulation in Developing Chloroplasts Disentangled

**DOI:** 10.1371/journal.pgen.1006173

**Published:** 2016-07-14

**Authors:** William Zerges

**Affiliations:** Biology Department, Concordia University, Montreal, Québec, Canada; Institut de Biologie Physico-Chimique, FRANCE

Distant cousins of photosynthetic bacteria reside in plant cells where, as organelles called plastids, they give color to fruit and flowers, make starch in roots, and carry out photosynthesis in leaves. Plastids acquire these tissue-specific functions during plant development by undergoing programed differentiation from totipotent proplastids of meristematic tissues. Plastid differentiation involves the regulated expression of the 100–200 genes of the plastid genome, which have been retained from the ancestral cyanobacterium that founded the plastids by endosymbiosis 1–2 x 10^9^ years ago [1]. Plastid genes are expressed by bacterial-type transcription and translation systems, which plastids also have retained during evolution [2]. The differentiation of chloroplasts, the photosynthetic plastid in green tissues, requires expression of plastid genes encoding polypeptide subunits of the photosynthesis apparatus and components of the organellar gene expression system. Photosynthesis subunits are produced in precise stoichiometries which, in the green alga *Chlamydomonas reinhardtii*, are brought about by feedback controls at the level of translation [3]. In C4 plants, distinct chloroplast types develop in mesophyll and bundle sheath cells through, in part, differential expression of the plastid genome. However, surprisingly little is known about how plastid genes are regulated during the transformation of proplastids into chloroplasts in the context of leaf development.

Developmental regulation of gene expression in plants is difficult to characterize. Young plants have many cell types in the gamut of developmental stages, and in many plant species, developing cells are in arrangements that complicate the physical dissection of tissues in specific stages for analyses. An excellent experimental system is provided by seedling leaves of the grasses, e.g., maize, rice, and barley. Each leaf has a developmental gradient along its long axis, with nonphotosynthetic meristematic cells (and proplastids) at the base and differentiated photosynthetic cells (and chloroplasts) at the tip (Fig 1) [4]. Analysis of the abundance of a subset of plastid mRNAs along this leaf gradient in maize and barley has revealed programmed changes in mRNA levels [5,6,7]. However, the degree to which mRNA levels predict the actual protein output of each gene has been unclear because of lack of data on translation rates.

**Fig 1:**
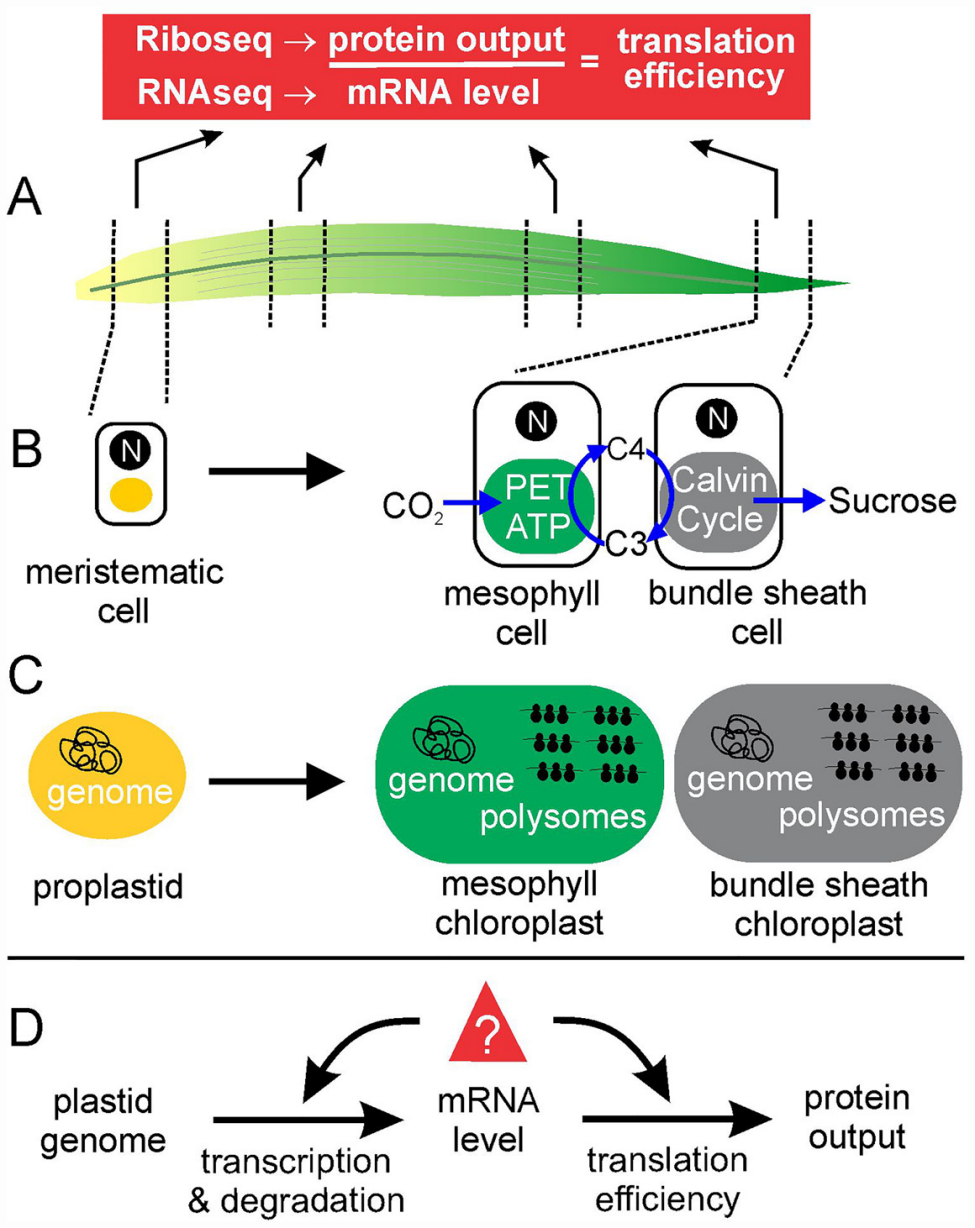
Plastid transcriptome and translatome profiles during chloroplast development in the maize seedling leaf reveal developmental patterns of plastid gene expression and the relative contributions of mRNA level and translational control in establishing them [8]. (A) The maize seedling leaf has a developmental gradient along the long axis. Dissected leaf segments (broken lines) and isolated mesophyll and bundle sheath cells (B) were used to profile the plastid transcriptome and translatome by next-generation RNA sequencing (RNAseq) and ribosome profiling (Riboseq), respectively. The developmental gradient has meristematic (undifferentiated) cells at the base (left) and mature photosynthetic cells at the tip (right), with their nuclei (N) and chloroplasts (green and grey). Mesophyll chloroplasts are specialized in the uptake of CO_2_, the photosynthetic electron transport (PET), ATP synthesis, and the synthesis of the C4 compound malate, which is transported to bundle sheath cells in a carbon concentrating system. Bundle sheath chloroplasts recover the CO_2_ (generating a C3 compound to be returned to mesophyll cells) and use it as substrate for the Calvin cycle and the subsequent synthesis of sucrose. (C) Proplastids and the chloroplasts have the plastid genome and gene expression system, including polysomes composed of translating ribosomes on mRNAs, for the synthesis of polypeptides. (D) The results of RNAseq and Riboseq analyses address the steps of plastid gene expression that determine protein output rate for each gene of the plastid genome.

Chotewutmontri and Barkan report in this issue [8] plastid transcriptome and translatome profiles at several points during the proplastid to chloroplast transition in young maize leaves, thereby revealing developmental patterns of protein production from each plastid gene and the relative contributions of mRNA level and translation rate in establishing them (Fig 1). They first optimized the ribosome profiling or “Riboseq” method for analysis of the plastid translatome in leaf tissue and in separated bundle sheath and mesophyll cells. Riboseq uses deep sequencing to tally footprints of translating ribosomes on specific mRNAs, revealing ribosome numbers and their precise locations on all mRNAs [9]. The abundance of ribosome footprints from each gene (normalized to mRNA length) serves as a proxy for the overall rate of protein synthesis from that gene. By comparing the Riboseq data to RNAseq data from the same tissue samples, the authors calculated relative translation efficiencies (i.e., ribosome footprint abundance divided by mRNA abundance). They describe the profiles of mRNA abundance, translational efficiency, and protein output for all plastid genes as a function of developmental stage (Fig 1).

The major importance of this work is the deciphering of the contributions of mRNA abundance and translational efficiency in the expression of the plastid genome during the proplastid to chloroplast transition. In this context, the results support five major conclusions. First, during chloroplast development, protein output from most plastid genes increases early and declines later. Changes in mRNA level account for many of these changes, but they are further refined by changes in translational efficiency. Second, two translational regulons are revealed, which are characterized by distinct developmental profiles at the level of translational efficiency during the proplastid to chloroplast transition. One regulon exhibits prioritized translation early in development and consists primarily of mRNAs encoding components of the chloroplast gene expression machinery. The other regulon exhibits peak translational efficiency later in development and consists primarily of mRNAs encoding photosynthesis proteins. Third, in bundle sheath and mesophyll chloroplasts, different patterns of protein output reflect primarily differences in mRNA abundance, although these are amplified in several cases by differences in translational efficiency. Fourth, photosynthesis complex subunit stoichiometry is reflected by relative rates of protein synthesis, and this tuning is programmed by a balancing of translational efficiencies with mRNA abundance. Fifth, the editing of plastid mRNAs, a process that changes specific C to U residues, generally is not a prerequisite for their translation. In addition, readers will find in the wealth of data something relevant to their particular interests in plastid gene expression.

Future research will use the groundwork and methods of Chotewutmontri and Barkan to advance further our understanding of plastid gene expression in development. One likely outcome is the discovery of developmental regulatory functions of known translation factors in the chloroplasts [10,11]. Also of interest will be to expand the analyses to the transcriptome and translatome of the nuclear-cytoplasmic compartments—for example, to describe how expression of nucleus-encoded and plastid-encoded subunits of photosynthetic complexes is coordinated during development. This work also opens avenues to explore relationships between translation and the polycistronic organization of plastid genes, mRNA splicing, the repair of photosynthesis complexes, and acclimation to changing environmental conditions. The disentangling of our understanding of the regulated steps in plastid gene expression in chloroplast development has begun.

## References

[1] Gould SB, Waller RF, McFadden GI (2008) Plastid Evolution. Annual Review of Plant Biology 59: 491–517. 10.1146/annurev.arplant.59.032607.092915 18315522

[2] Sun Y, Zerges W (2015) Translational regulation in chloroplasts for development and homeostasis. Biochim Biophys Acta 1847: 809–820. 10.1016/j.bbabio.2015.05.008 25988717

[3] Choquet Y, Wollman FA (2009) The CES Process. In: Harris EH, Stern DB, Witman GB, editors. The Chlamydomonas Sourcebook (Second Edition). Oxford: Elsevier pp. 1029–1066.

[4] Leech RM, Rumsby MG, Thomson WW (1973) Plastid differentiation, acyl lipid, and Fatty Acid changes in developing green maize leaves. Plant Physiol 52: 240–245. 16658539 10.1104/pp.52.3.240 PMC366477

[5] Börner T, Aleynikova AY, Zubo YO, Kusnetsov VV (2015) Chloroplast RNA polymerases: Role in chloroplast biogenesis. Biochimica et Biophysica Acta (BBA)—Bioenergetics 1847: 761–769. 25680513 10.1016/j.bbabio.2015.02.004

[6] Baumgartner BJ, Rapp JC, Mullet JE (1989) Plastid Transcription Activity and DNA Copy Number Increase Early in Barley Chloroplast Development. Plant Physiology 89: 1011–1018. 16666609 10.1104/pp.89.3.1011 PMC1055959

[7] Cahoon AB, Takacs E, Sharpe R, Stern D (2008) Nuclear, chloroplast, and mitochondrial transcript abundance along a maize leaf developmental gradient. Plant Molecular Biology 66: 33–46. 17932771 10.1007/s11103-007-9250-z

[8] Chotewutmontri P, Barkan A (2016) Dynamics of chloroplast translation during chloroplast differentiation in maize. PLoS Genet 12(7): e1006106. 27414025 10.1371/journal.pgen.1006106 PMC4945096

[9] Ingolia Nicholas T (2016) Ribosome Footprint Profiling of Translation throughout the Genome. Cell 165: 22–33. 10.1016/j.cell.2016.02.066 27015305 PMC4917602

[10] Tourasse NJ, Choquet Y, Vallon O (2013) PPR proteins of green algae. RNA Biol 10: 1526–1542. 10.4161/rna.26127 24021981 PMC3858436

[11] Belcher S, Williams-Carrier R, Stiffler N, Barkan A (2015) Large-scale genetic analysis of chloroplast biogenesis in maize. Biochimica et Biophysica Acta (BBA)—Bioenergetics 1847: 1004–1016. 25725436 10.1016/j.bbabio.2015.02.014

